# Breast Cancer Environment Centers and Advocacy

**DOI:** 10.1289/ehp.1103466

**Published:** 2011-05

**Authors:** Mary S. Wolff, Janice Barlow

**Affiliations:** Mount Sinai School of Medicine, New York, New York, E-mail: mary.wolff@mssm.edu; Zero Breast Cancer, San Rafael, California

As Breast Cancer and Environment Research Center (BCERC) project leaders, we would like to address what we believe represents inaccuracies and omissions in the recent article by Baralt and McCormick (2010). Using self-citations, the authors asserted that genes and environment were not included in breast cancer research before advocacy efforts emerged. Yet the environment has long been implicated in breast cancer etiology; for example, for > 50 years the laboratory model of mammary carcinogenesis has involved administration of environmental chemicals (Medina 2007). Further, the Long Island Breast Cancer Study Project (LIBCSP) was not the first environment–breast cancer grant, as suggested by Baralt and McCormick. The National Institute of Environmental Health Sciences issued such grants as early as 1991, including “Environmental Factors and Breast Cancer in High-Risk Areas” [Request for Applications (RFA) CA/ES-93-024] in 1993.

The LIBCSP has been enormously productive, continuing even now, with > 100 scientific publications and $21 million in grant funding using LIBCSP resources. Baralt and McCormick’s (2010) criticism of the LIBCSP ignores the rigorous review process of National Institutes of Health grants, requiring an undeniable hypothesis, scientific plausibility, and high probability of success. What Baralt and McCormick described is the dissatisfaction of some (but not all) advocates with that research process during the initial years of the LIBCSP.

Baralt and McCormick (2010) used the word “frustration*”* 16 times, without noting the impressive contributions of the BCERC Community Outreach and Translation Cores (COTC) projects. Advocacy and COTC in the BCERC since 2003 have resulted in extensive and innovative dissemination of knowledge and new ideas (Breast Cancer and the Environment Research Program 2011). Mutual learning was facilitated by the participation of advocates and research staff in weekly staff meetings, monthly epidemiology and COTC calls, 16 subcommittee meetings and calls, and organizing calls for the biannual meetings. Coordinated COTC, advocate, and scientific sessions were part of the biannual BCERC meetings. Rather than “frustration,” the past 7 years could be better summarized as an ongoing, interactive, collaborative, critical process of science and advocacy–indeed a new paradigm of scientific method.

As noted by Baralt and McCormick (2010), the 2002 RFA for BCERC did not require adherence to principles of community-based participatory research. The BCERC COTC members represented a range of experience in community-based participatory research; few had training in basic science. Each center developed different COTC models of community involvement and engagement, not included by Baralt and McCormick in their article. The Bay Area COTC incorporated the principles of community-based, participatory research and used those principles to evaluate the extent to which the approach was participatory and to ascertain the benefits and challenges of the participatory aspects of the project as perceived variously by community, advocacy, and scientific partners (Van Olphen et al. 2009). Other centers used quite different models of community engagement and, accordingly, should be evaluated in a different fashion. Thus, it would have been appropriate for Baralt and McCormick (2010) to assess which model most effectively met the aims stated in the 2002 RFA. Another difference between centers was that, except for the Bay Area, the COTCs were part of a research or academic institution. Thus, we faced multiple challenges on how to effectively involve communities and advocates in research. Over the first 7 years, centers developed a continuum of strategies to create partnerships with the basic scientists and epidemiologists involved in BCERC.

Baralt and McCormick (2010) omitted important details describing their methodology from the article. Specifically, in their Table 1 they included demographics about the sex and race/ethnicity of the investigators from BCERC centers, but no similar table characterized the participants in their study. [The Bay Area BCERC COTC included an African-American member, not four whites as Baralt and McCormick showed in their Table 1.] In addition, the authors did not discuss the involvement of advocates compared with nonadvocates in activities of COTCs at the four centers. It was unclear whether survey participants included only scientists and advocates formally connected with the centers (e.g., those listed in their Table 1) or if they included non-BCERC scientists and advocates who attended the conferences. Also, if the respondents in 2005 and 2007 were completely different, as suggested, it was not appropriate to pool the data nor to report any changes over time. We support advocate participation in research, and we recognize that methods for quantifying their contributions require unique approaches.
